# Case Report: Kyrieleis plaques in an unusual Behcet's disease uveitis

**DOI:** 10.3389/fmed.2025.1544319

**Published:** 2025-05-22

**Authors:** Jiani Li, Tantai Zhao, Xiaojian Guo, Yangyan Xiao

**Affiliations:** ^1^Department of Ophthalmology, The Second Xiangya Hospital, Central South University, Changsha, Hunan, China; ^2^Department of Ophthalmology, Hunan University of Medicine General Hospital, Huaihua, Hunan, China

**Keywords:** Behcet's disease, Kyrieleis plaques, central serous chorioretinopathy, uveitis, adalimumab

## Abstract

Kyrieleis plaques are a rare retinal manifestation that has rarely been reported in Behcet's disease uveitis. We report the case of a 41-year-old man who presented with Behçet's disease uveitis combined with central serous chorioretinopathy. Laboratory studies showed positive for human leukocyte antigen B51. Polymerase chain reaction analysis of aqueous humor excluded infectious uveitis. Kyrieleis plaques were observed in the bilateral retinal arteries. These plaques persisted in both eyes after half a year of immunosuppressive treatments. Kyrieleis plaques are not limited to infectious uveitis; they can also be associated with Behçet's disease uveitis.

## Introduction

Kyrieleis plaques are a rare, non-specific retinal manifestation that presents as focal or segmental yellowish-white deposits within retinal arteries (1). These plaques, which indicate severe intraocular inflammation, are usually observed in infectious uveitis (2). Behcet's disease, a multisystem vasculitis, is characterized by recurrent oral ulcers, ocular inflammation, genital ulcers, and skin lesions (3). Uveitis is the predominant manifestation of ocular involvement in Behçet's disease, which can cause irreversible vision loss (4). Kyrieleis plaques have rarely been reported in Behcet's disease uveitis (5). This case represents bilateral Kyrieleis plaques in a patient presenting with Behçet's disease uveitis complicated by central serous chorioretinopathy (CSC).

## Case report

**Figure 1:**
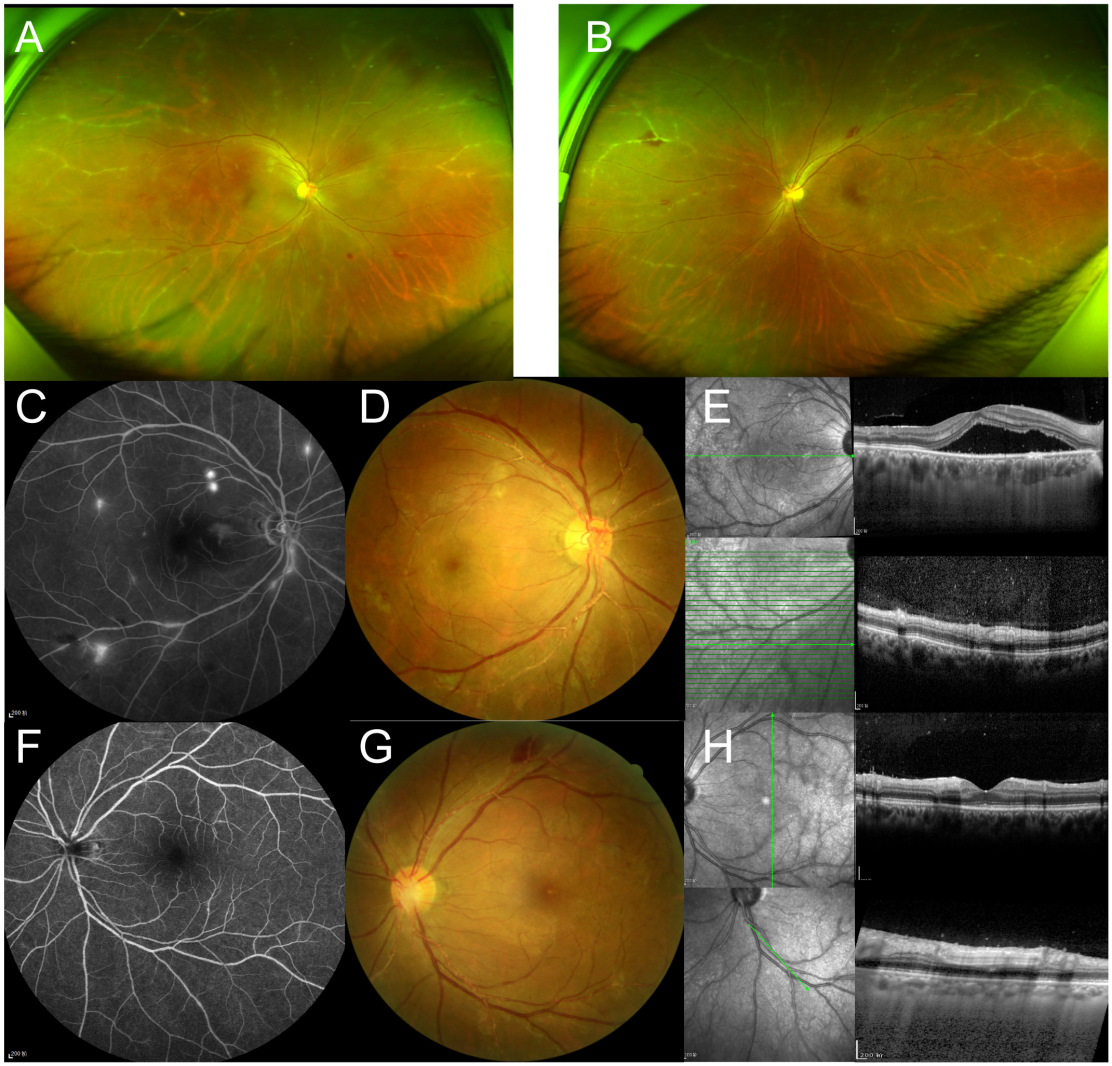
**(A, B)** Optos ultra-widefield retinal imaging shows whitening changes in the peripheral arterioles bilaterally. **(C, D)** Fundus/fluorescein angiography of the right eye reveals vessel leakage and macular smoke-stack hyperfluorescence. **(F, G)** The arterial plaques show no filling defects or leakage of fluorescein dye in the left eye. **(E, H)** OCT shows the hyperreflective changes in the vessel wall along with the arterial plaques and hyperreflective dots in the vitreous cavity bilaterally. Serous neuro-retinal detachment and pachychoroid changes are observed in the right eye.

A 41-year-old Asian man presented to the ophthalmology clinic with a history of bilateral red eye for 3 weeks and decreased vision for 2 weeks, particularly in his right eye. He reported experiencing recurrent oral ulcerations three times a month for the past 3 years and had a previous cerebral infarction without residual deficits 5 years ago. This patient had no family history. The best-corrected visual acuity (BCVA) was 20/50 in the right eye and 20/33 in the left eye, with normal intraocular pressure bilaterally. On ophthalmic examination, both eyes had 2+ cells and 2+ flare in the anterior chamber but no synechiae. In both eyes, funduscopic examination revealed vitritis, retinal hemorrhages, and focal segmental yellowish-white plaques along the arterioles (Figures 1A, B, D, G).

Fluorescein angiography displayed vessel leakage with retinal hemorrhages, no leakage from vessels with plaques in both eyes, and a smoke-stack pattern of hyperfluorescence in the right macula (Figures 1C, F). Optical coherence tomography (OCT) showed hyperreflectivity of the vessel wall in the plaque area in both eyes and macular retinal detachment in the right eye (Figures 1E, H). Laboratory testing revealed positivity for human leukocyte antigen B51 (HLA-B51). All other results, including erythrocyte sedimentation rate, C-reactive protein, human leukocyte antigen B27, antinuclear antibody, and anti-citrullinated protein antibody, were within normal limits. Polymerase chain reaction analysis of aqueous humor excluded herpes zoster virus, varicella-zoster virus, cytomegalovirus, and Epstein–Barr virus infection. Lung computed tomography and color Doppler echocardiography were both normal. Atherosclerotic plaque formation was shown in the left subclavian artery using arterial ultrasonic Doppler. Skin allergy tests were performed, and the results showed a positive finding.

**Figure 2:**
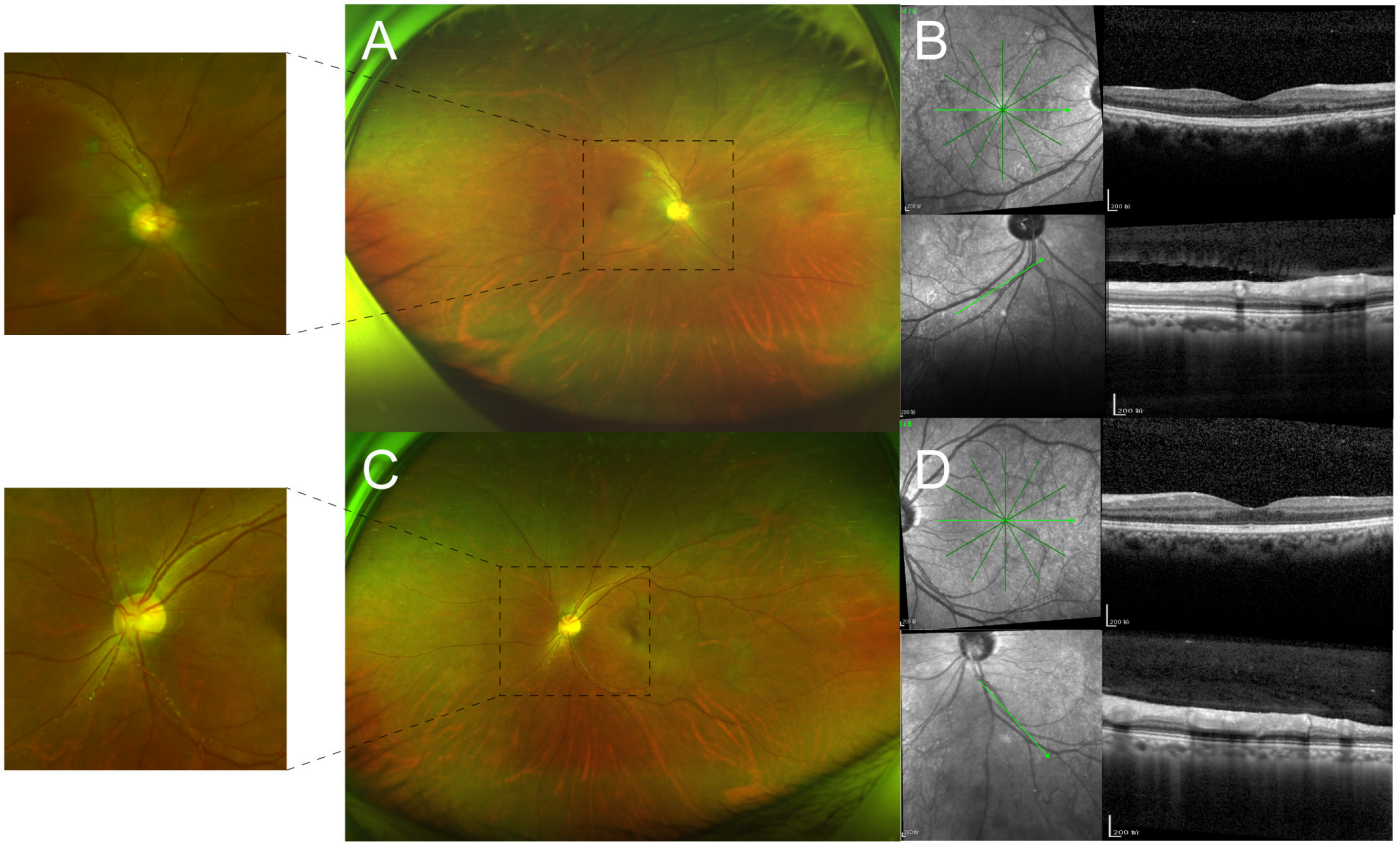
**(A, C)** Optos ultra-widefield retinal imaging reveals that the plaques partially persisted in both eyes after half a year of follow-up. **(B, D)** OCT shows the disappearance of hyperreflective dots in the vitreous cavity and complete absorption of subfoveal fluid.

Behcet's disease was diagnosed using the International Study Group criteria (6). In this case, funduscopic examination presented with pachychoroid changes and retinal detachment, which were confirmed and characterized as CSC by OCT (Figures 1D, E). Fluorescein angiography displayed a smoke-stack pattern of hyperfluorescence in the right macula, the characteristic appearance of CSC (Figure 1C). The patient was treated with adalimumab 40 mg every other week, cyclophosphamide 8 mg/kg once a week, thalidomide 50 mg daily, and methylprednisolone 24 mg daily with gradual reduction. With the prednisone decreased to 6 mg in 2 months, the subfoveal fluid of the right eye was still the same. Hence, a subthreshold micropulse laser was used. After half a year of follow-up, BCVA improved to 20/20 in each eye. OCT showed the disappearance of hyperreflective dots in the vitreous cavity, as well as complete absorption of subfoveal fluid (Figures 2B, D). Kyrieleis plaques and abnormal fluorescein angiography features persisted in both eyes (Figures 2A, C).

## Discussion

Kyrieleis plaques, also known as segmental retinal arteritis, have primarily been described in association with infectious uveitis, such as *Toxoplasmosis chorioretinitis, Mycobacterium tuberculosis, Treponema pallidum*, Herpes Zoster virus, and Varicella Zoster virus infections (1, 7). With a glistening appearance and resembling calcific lesions, these plaques were initially proposed as arteriosclerosis (8). Then, it has been suggested that they are exudates or immune cells migrating from an adjacent vessel and depositing on the vessel wall (9). Based on multimodal imaging techniques, Pichi et al. found that indocyanine green angiography of Kyrieleis plaques showed focal early hypofluorescence and late hyperfluorescence, while fluorescein angiography of Kyrieleis plaques showed early hypofluorescence and intermediate hyperfluorescence. Due to the different molecular characteristics between indocyanine green and fluorescein, they first speculated that these plaques represent inflammatory involvement within the endothelial cells of vessel walls rather than periarterial or endoluminal injury (10). In addition, OCT scans revealed hyperreflectivity of the arterial walls at Kyrieleis plaque sites, with no evidence of lumen obstruction or fluorescein dye leakage on fluorescein angiography (10). Recently, Milella et al. used adaptive optics to describe these lesions, which showed segmental hyporeflectivity confined to the vessel walls. This study further supports the view that Kyrieleis plaques are characterized by an inflammatory involvement within the vascular endothelium (11).

Behcet's disease, a chronic inflammatory disorder involving systemic vasculitis, is characterized by recurring symptoms such as oral aphthous ulcers, genital lesions, uveitis, and cutaneous eruptions (3). Genetic factors and environmental triggers play a crucial role in the pathogenesis of Behcet's disease (12). HLA-B51 has been considered the strongest predisposing gene for BD. In the Japanese diagnostic criteria, HLA-B51 is considered a valuable finding in cases with no strong diagnostic implications (13). In addition, there is growing evidence that HLA-B51 affects clinical phenotypes of Behçet's disease (14). Horie et al. reported that HLA-B51-positive Behcet's disease patients have a higher risk of eye complications (15). Another study also found that HLA-B51 positive is a risk factor for ocular lesions (16).

Uveitis is one of the most common ocular manifestations of Behcet's disease. Ocular involvement may cause severe tissue damage and lead to poor prognosis (4). There has only been one reported case of coexistent Kyrieleis plaques and ocular Behcet's disease (5). This case showed multimodal imaging of Kyrieleis plaques in the coexistence of Behcet's disease uveitis and CSC, which makes our case even more unique. Kyrieleis plaques are predominantly unilateral and usually occur in the area of active retinal infection or inflammation (2). Kyrieleis plaques are rare events and need to be distinguished from arterial emboli, frosted branch angiitis, and vascular sheathing. They are not intraluminal arterial emboli. They only affect the retinal arteries, while frosted branch angiitis affects both the retinal arteries and veins. Fluorescein angiography is an important tool for identifying them (17). These plaques have not been found to worsen the clinical prognosis of ocular lesions, and they may persist or disappear as ocular inflammation is treated (2, 10). Kyrieleis plaques persist even following the resolution of acute inflammation, as observed in our case.

Behcet's disease uveitis is characterized by relapse and remission in the course of the disease. The primary therapeutic objective of Behçet's disease uveitis is to control disease activity and prevent relapse in the long term (4). Corticosteroid therapy, which includes both topical and oral corticosteroids, is the most frequently used regimen. Immunosuppressive treatment approaches were often used and recommended, including azathioprine, cyclosporine-A, and interferon-alpha (3). However, biologic agents, particularly monoclonal antibodies to TNF-α, such as infliximab and adalimumab, have been shown to be effective in treating Behçet disease uveitis (18). In this case, the patient had Behçet's disease uveitis combined with CSC. CSC is characterized by neuroretinal detachment due to the presence of serous subretinal fluid, which can result in irreversible retinal pigment epithelium and photoreceptor damage (19). Corticosteroids have been described to be an independent risk factor for CSC (20). Thus, a subthreshold micropulse laser was used to increase fluid resolution (21). Adalimumab, a correct alternative treatment, was used to control disease activity and prevent relapses of Behçet's disease in the long term (18).

## Conclusion

This case showed multimodal imaging of Kyrieleis plaques along with Behcet's disease uveitis and CSC. In our case, we would like to emphasize that Kyrieleis plaques, a non-specific retinal manifestation, may be observed in Behcet's disease uveitis.

## Data Availability

The original contributions presented in the study are included in the article/supplementary material, further inquiries can be directed to the corresponding author.
